# Unlocking the therapeutic potential of antibody–drug conjugates in targeting molecular biomarkers in non-small cell lung cancer

**DOI:** 10.1186/s43046-025-00264-4

**Published:** 2025-03-03

**Authors:** Shruti Soni, Kaivalya Megha, Vraj B. Shah, Aayushi C. Shah, Shelly Bhatt, Manthan Merja, Avinash Khadela

**Affiliations:** 1 Department of Pharmacology L. M. College of Pharmacy, Navrangpura, Ahmedabad, Gujarat 380009 India; 2 Starlit Cancer Centre, Kothiya Hospital campus, Ahmedabad, Gujarat 382350 India; 3 Present address: L. M. College of Pharmacy, Navrangpura, Ahmedabad, Gujarat 380009 India

**Keywords:** Non-small cell lung cancer, Antibody–drug conjugates, Clinical biomarkers, Precision oncology, Targeted therapy, Immunotherapy

## Abstract

Non-small cell lung cancer (NSCLC) is a prevalent and lethal malignancy worldwide, posing significant challenges to patient survival. Recent advancements in the field of oncology have introduced immunotherapy and targeted therapy as primary treatment modalities for NSCLC. However, the emergence of treatment resistance and relapse has impeded their long-term effectiveness. Antibody–drug conjugates (ADCs), a rapidly evolving class of anti-cancer agents, offer a promising solution to this issue by harnessing the specificity of monoclonal antibodies and the cytotoxic potency of drug payloads. ADCs have demonstrated notable potential in targeting both highly expressing and low-expressing malignant cells, with early-phase clinical trials yielding superior survival outcomes in NSCLC patients. This review comprehensively outlines the recent advancements in ADC-based strategies for managing NSCLC, supported by evidence from clinical trials. Additionally, the review delves into the oncogenic mechanisms of various biomarkers and offers insights into strategies for their detection in NSCLC patients. Lastly, a forward-looking perspective is provided to address the challenges associated with the utilization of ADCs in NSCLC therapy.

## Introduction

The last few decades have witnessed remarkable developments in cancer diagnostics and treatment strategies. Identification of oncogenic drivers and the development of targeted therapeutic agents have shifted the paradigm of cancer care. This phenomenon of the molecular profiling of tumor cells to identify the oncogenic alterations is widely known as precision oncology [1]. Non-small cell lung cancer (NSCLC) has been exemplified as the prototype disease for precision oncology in solid tumors. This was the result of the identification of epidermal growth factor receptor (EGFR) mutations which led to the inclusion of EGFR tyrosine kinase inhibitors (TKIs) into the treatment regimen of patients with EGFR mutant NSCLC [2].

The NSCLC is a heterogeneous malignancy involving a large number of oncogenic driver alterations. NSCLC accounts for nearly 85% of lung cancer cases and is further divided into adenocarcinoma (NSCLC-AC), squamous cell carcinoma (NSCLC-SCC), and large cell carcinoma [3]. More than half of the cases of NSCLC are diagnosed at an advanced stage and suffer from poor prognostic outcomes with a 5-year survival rate of only 2–20% [4]. Currently, targeted therapeutic agents are considered the standard of care for most NSCLC patients with molecular alterations [5]. For patients without any actionable oncogenic mutations, immunotherapy with programmed cell death ligand-1 (PD-1/PD-L1) and cytotoxic T-lymphocyte-associated protein 4 (CTLA-4) is utilized as the treatment of choice [6]. Nevertheless, the majority of patients treated with immunotherapy or targeted therapy agents develop resistance or relapse following a few years of treatment and require alternative therapeutic strategies, necessitating the need for novel treatment approaches [7].

**Fig. 1:**
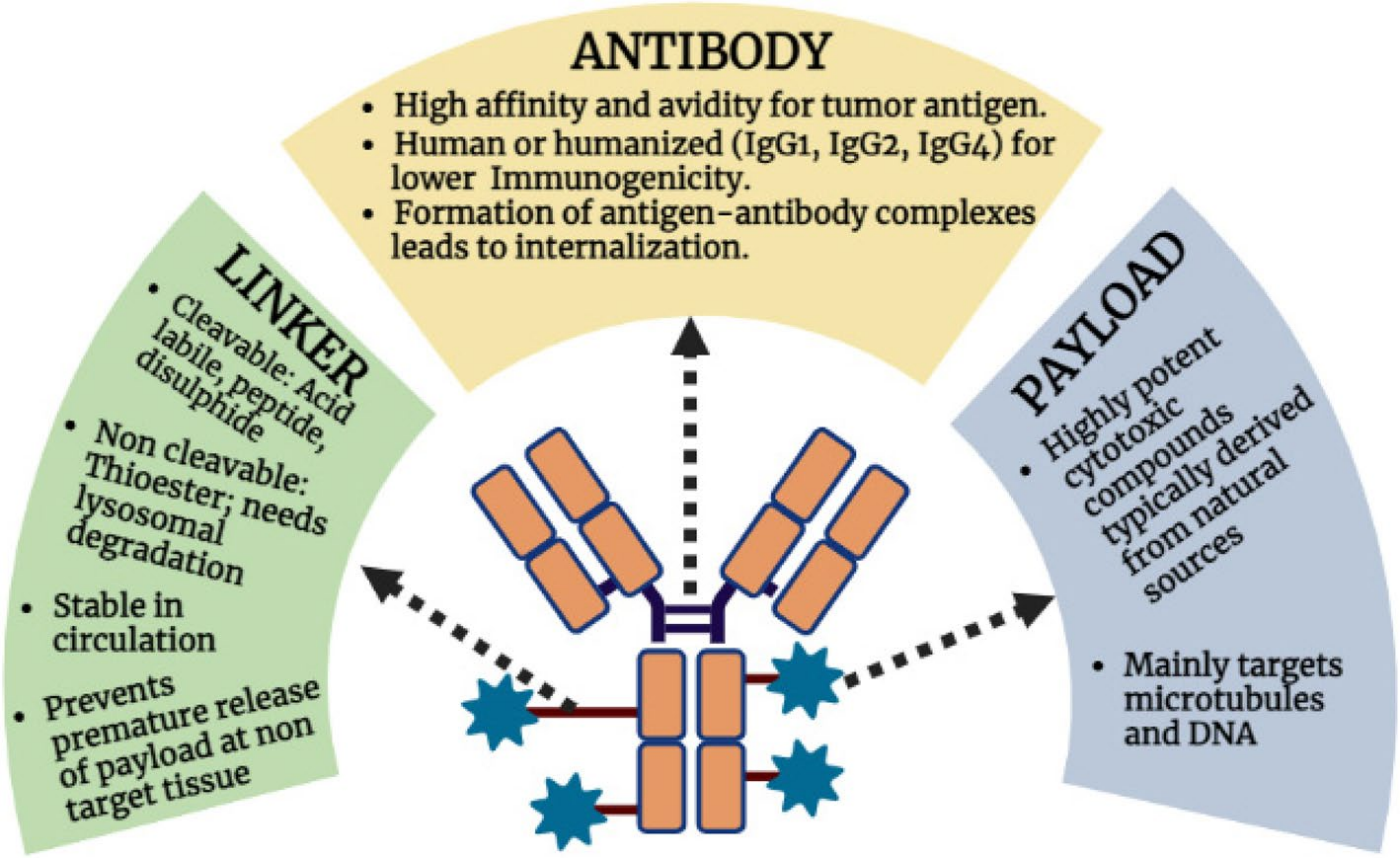
Structural bifurcation of ADCs along with their properties. Immunoglobulin (Ig), Deoxyribonucleic acid (DNA)

**Fig. 2:**
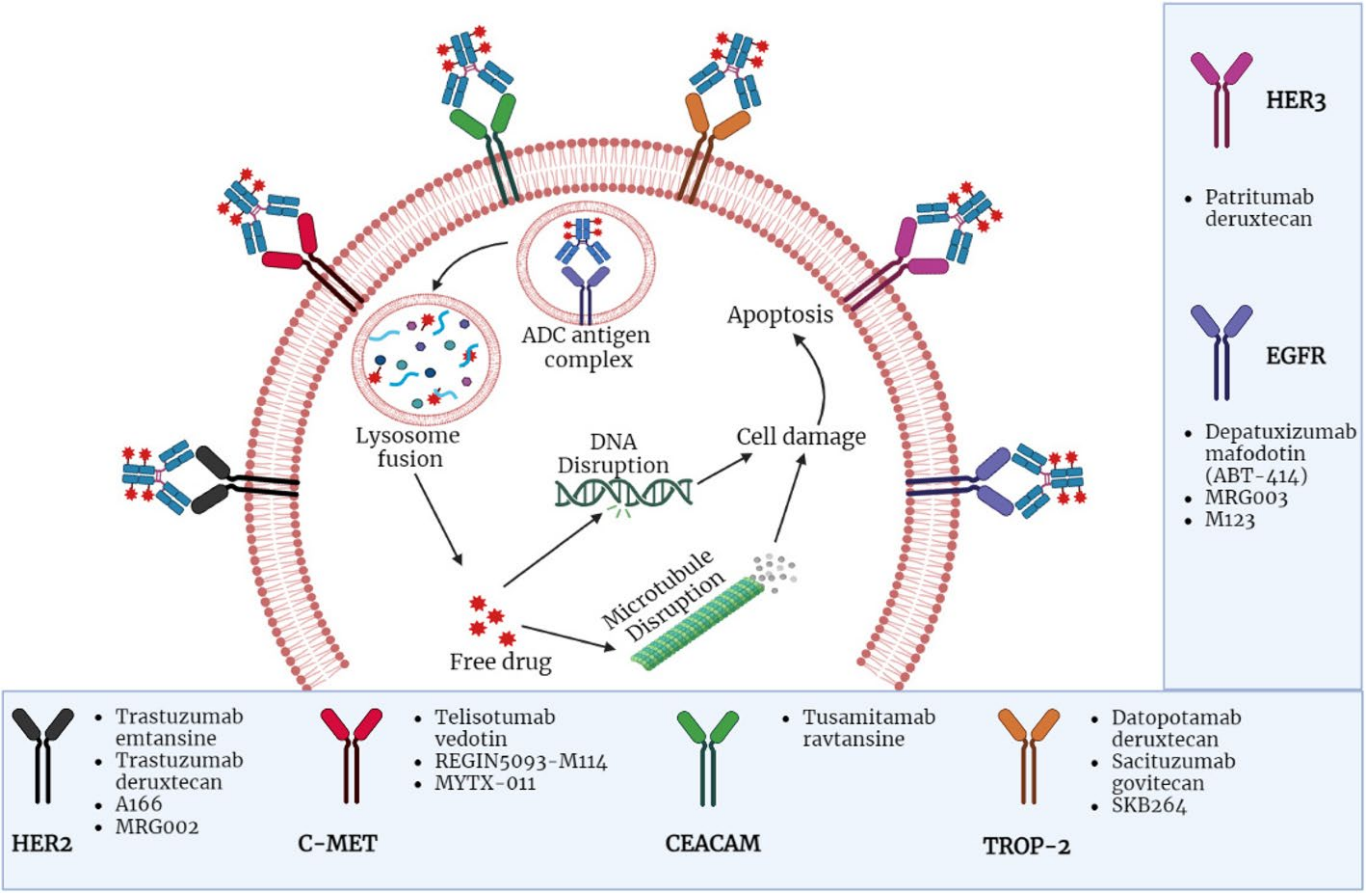
Summary of different oncogenic biomarkers and effective antibody–drug conjugates in patients suffering from non-small cell lung cancer

Antibody–drug conjugates (ADCs) are a unique therapeutic ensemble combining the target selectivity of monoclonal antibodies (mAb) with the cytotoxic effects of chemotherapeutic agents [8]. In order to assemble a safe and efficacious ADC, a meticulous selection of antibodies, payload, and the connecting linker moiety is essential [9]. The structural bifurcation and properties of each component of an ADC have been provided in Fig. 1. The mechanism of action of ADCs is multifaceted, involving several steps (Fig. 2). Firstly, the mAb binds with the target antigen exclusively present in the cancer cell, in order to reduce the off-target toxicities. This ADC-antigen complex is then internalized into the cell followed by lysosomal fusion and cytotoxic drug release into the cell. The cytotoxic payload of an ADC comprises microtubule disrupting agents, topoisomerase inhibitors, or DNA cleavage agents, which are responsible for the resultant cytotoxicity [10]. The structural layout of various ADCs currently under investigation in NSCLC is summarized in Table 1. In addition to the cytotoxic effects exerted on the antigen-positive tumor cells, the metabolites of cytotoxic agents might permeate through the membrane and kill adjacent tumor cells by their bystander-killing effects. This anomaly can offer a significant advantage when a target heterogenicity is observed within the tumor microenvironment (TME) and not all tumor cells sufficiently express the target antigen [11]. ADCs have successfully paved their way into the therapeutic regimen of several malignancies, including breast cancer and gastric cancers following their Food and drug administration (FDA) approvals. Numerous ADCs are currently under development in lung cancer patients with promising results from phase I–II clinical trials [12].

**Table 1:** Structural layout of ADCs currently under development in NSCLC

Maker	ADCs targeting that marker	Drug	Antibody	Linker
EGFR	Depatuxizumab mafodotin	MMAF	Depatuxizumab	Non-cleavable linker maleimidocaproyl (mc)
	MRG003	MMAE	EGFR-specific IgG1 antibody	Protease cleavable valine-citrulline (vc)
	M1231	Hemiasterlin moiety	Anti-MUC1/EGFR bispecific antibody	Cleavable valine-citrulline
HER2	Trastuzumab–emtansine	Derivative of maytansine	Trastuzumab	N-succinimidyl-4-(N-maleimidomethyl) cyclohexane-1-carboxylate
	Trastuzumab deruxtecan	Deruxtecan	Trastuzumab	Cleavable tetrapeptide-based linker
	A166	Duostatin-5	Trastuzumab	Protease cleavable linker
	MRG002	MMAE	Anti HER-2 IgG1 mAb	Valine citrulline linker
HER3	Patritumab Deruxtecan	MAAA-1181a	Patritumab	Tetrapeptide-based cleavable linker
c-MET	Telisotuzumab vedotin	MMAE	IgG1–kappa ABT-700	Cleavable maleimidocaproyl-valyl-citrullinyl-p-aminobenzyloxycarbonyl (mc-val-cit-PABC)
	REGN5093-M114	Maytansine derivative M24	MET-targeting human IgG4p bispecific antibody	–
	MYTX-011	MMAE	pH-dependent anti-cMET antibody	–
TROP-2	Datopotamab deruxtecan	–	Humanized anti-TROP2 monoclonal antibody	Tetrapeptide-based linker
	SKB264	Proprietary cytotoxic, belotecan-derived payload	Anti-Claudin 18.2 antibody	–
	Sacituzumab govitecan	SN38, Irinotecan active metabolite	IgG1-kappa	Carbonate
CEACAM -5	Tusamitamab ravtansine (SAR408701, tusa	Maytansinoid DM4	CEACAM5 targeting antibody	SPDB linker [N-succinimidyl 4-(2-pyridyldithio)butanoate

*MMAF* monomethyl auristatin F,* EGFR* epidermal growth factor receptor, *c-MET* c-Mesenchymal-epithelial transition factor-1, *HER-2* human epidermal growth factor-2, *MMAE* monomethyl auristatin E, *Ig* immunoglobulin

This review presents an elaborate discussion regarding the utilization of ADCs in NSCLC. Numerous biomarkers responsible for the development of NSCLC along with their oncogenic mechanisms have been discussed thoroughly. This review focuses on the respective ADCs working on those specific biomarkers in NSCLC along with the evidence retrieved from the clinical trials.

### Promising ADC targets for the treatment of NSCLC

#### c-MET

c-Mesenchymal-epithelial transition factor-1 (c-MET) is a transmembrane tyrosine kinase receptor and an oncogenesis driver explored as a potential target by anti-cancer moieties for delivering anti-tumor activity [13, 14]. Alternatively termed as Hepatocyte growth factor (HGF) receptor, it encodes a protein tyrosine kinase which holds responsibility for regulating important cellular processes namely cell differentiation, cell proliferation, cell division, movement, and apoptosis. HGF expression is carried out by stromal and mesenchymal cells and is a paracrine signaling molecule which solely acts as a ligand for c-MET [15]. In tumor cells, the activation of MET occurs in multiple ways including receptor and ligand overexpression, gene amplification, gene mutation, and rearrangements in chromosomes. Two primary forms of MET expression showing a potential association with the progression of NSCLC are (1) MET exon 14 skipping alteration and (2) MET gene copy number gain (CNG) [16]. Moreover, MET gene amplification is an identified culprit defined to induce resistance toward EGFR-targeted therapies [17]. MET alterations have the most tendency to be involved in NSCLC-AC courses while they are also prevalent in NSCLC-SCC. Nearly 5% of NSCLC patients show MET exon 14 skipping. On the contrary, a very low percentage of patients have MET gene amplification. MET exon 14 skipping and MET amplification both can be diagnosed through comprehensive next-generation sequencing (NGS) and fluorescence *in-situ* hybridization (FISH) [18]. The silencing of MET through small interference RNA or short hairpin RNA or inactivation of the biomarker through micro-RNAs has been stated as prominent in vitro for cell viability, downstream signaling, cell motility, migration, and invasion [19]. Extensive efforts have been made on MET inhibitors considering pre-clinical models to further lead a direction for clinical studies investigating these agents. Drawing evidence from a study exploring c-MET receptor expression in NSCLC cell lines, the methodology involved evaluation of c-MET receptor through the employment of western blot analysis [20]. 11 established cell lines and 104 surgically resected tissues were subjected to western blot where the cell line distribution consisted of 8 adenocarcinomas, 2 squamous cell carcinomas and a large cell carcinoma [21]. The NSCLC tissues used for the study were obtained from 104 patients, were frozen, and kept at − 80 °C. For western blotting, each NSCLC cell line was formerly harvested at the exponential growth phase. Immediately the cell pellet was homogenized in 1 ml of 10 mM Tris-HCl buffer [22]. The homogenate was later centrifuged at 10,000 rpm for 15 min at 4 °C and the clear supernatant, thus obtained, was used for western blotting. All cell lines affirmed a strong presence of c-MET proteinaceous bands of 145 kDa and 170 kDa. The clarity of results was found slightly lacking as two cell lines used in this study originated from negative c-MET expression also suggested its presence which may be justified as a consequence of cell line influence during cultivation. The conclusion of this clinicopathological study also revealed that NSCLC positive for c-MET expression may present worse outcomes as compared to NSCLC without c-MET involvement [23].

A conventional c-MET inhibitor such as small molecule TKIs delivers potential anti-tumor efficacy in several tumor cell lines expressing higher HGF concentrations over 50 ng/mL. The tumor cell lines expressing lower HGF levels in human serum ranging between 0.4 to 0.8 ng/mL remain undetected by c-MET inhibitors as the sensitivity of conventional therapies diminishes at such non-physiological concentrations [24]. On the other hand, these TKIs show limited implications on therapeutic regimens due to the emergence of acquired resistance.

For the detection of such lowly expressed c-MET biomarkers, the concept of ADCs presents an affirmative potential. A summary of different oncogenic biomarkers and ADCs targeting them in NSCLC patients has been provided in Fig. 2. The current drug ecosystem has developed three such ADCs targeting c-MET mutation and amplification phenomena.

Telisotuzumab vedotin (Teliso-V), formerly named ABBV-399, holds FDA approval for the treatment of advanced/metastatic stage of EGFR wild type, nonsquamous NSCLC positive for c-MET mutation [25, 26]. Following the encouraging data from the LUMINOSITY (NCT03539536) phase 2 trial, Telisotuzumab vedotin received breakthrough therapy designation (BTD) from the FDA for previously treated NSCLC with c-MET overexpression. This trial included 136 patients with advanced NSCLC who were further divided into cohorts based on their histopathological examinations (non-squamous [NSQ] or squamous [SQ], EGFR mutation status [wild-type or mutant]). The NSQ cohort further subdivided patients based on whether the c-MET expression was high or intermediate. The study is being carried out in two stages. Stage 1 was focused on identifying that which c-MET overexpressing NSCLC populations were most suitable for Telisotuzumab vedotin. The results demonstrated an overall response rate (ORR) of 36.5% in the NSQ EGFR-WT cohort (52.2% in the c-MET high and 24.1% in the c-MET intermediate population). The NSQ EGFR mutant and SQ cohort showed only modest improvements. Serious adverse events were seen in two patients from the SQ cohort in the form of sudden death and pneumonitis [27]. From this trial data, it can be concluded that the histopathological examinations play a crucial role in determining the response to the therapy as the NSQ cohort with WT-EGFR had the best response. Moreover, the c-MET intermediate population also showed improvements from the therapy, which demonstrates the potential of ADCs in targeting not only the high-expressing cells but also the moderate expression present in the target cells. Another phase 2 trial Lung-MAP S1400K evaluated the outcome of administering Tisotuzumab vedotin in patients with c-MET positive previously treated squamous cell lung carcinoma. Patients were divided into 2 cohorts based on their previous exposure to immune checkpoint inhibitors (ICIs). The patients received 2.7 mg/kg dose of Tisotuzumab vedotin every 3 weeks until the occurrence of unacceptable toxicities or disease progression. The results were disappointing as the median overall survival (OS) and progression-free survival (PFS) were 5.6 months and 2.4 months with only two responses reported in the immunotherapy naïve cohort. Additionally, two patients developed pneumonitis resulting in death. The trial was discontinued owing to the lack of efficacy which did not justify the further evaluation [28]. Thus, from the above two discussed studies it can be inferred that Tisotuzumab vedotin has failed to produce significant responses in the patients with squamous cell carcinoma. However, these results have not blocked further studies for Tisotuzumab vedotin in c-MET positive NSCLC patients as currently, many trials are under investigation which is expected to derive substantial outcomes soon. Evidence from the completed clinical trials evaluating the application of ADCs in NSCLC is summarized in Table 2.

**Table 2:** Clinical data on the use of ADCs in NSCLC

ADC	Trial	NCT no	Phase	Sample size	Tumor alteration	Clinical setting	Results	Remarks
Telisotuzumab vedotin	LUMINOSITY	NCT03539536	II	136	EGFR mutation (wild type or mutant), further subdivided into high or intermediate c-MET expression	Locally advanced/metastatic NSCLC	ORR 36.5%	Highest response to the therapy was seen in wild-type EGFR and c-MET intermediate cohorts
	Lung-MAP S1400K	NCT03574753	II	49	c-MET positive tumors	NSCLC-SCC previously treated with ICIs	mOS 5.6 months; mPFS 2.4 months	Trial was discontinued due to a lack of efficacy
Datopotamab deruxtecan	TROPION-PanTumor01	NCT03401385	I	175	–	Patients with solid tumors	DCR 87%; ORR 21%; mPFS 8.2 months	Dato-Dxd was found to have a manageable safety profile along with RP2D of 6 mg/kg
Sacituzumab govitecan	IMMU-132–01	NCT01631552	I/II	54	Patients were enrolled regardless of the TROP-2 level	Heavily pretreated metastatic NSCLC	ORR 16.7%; CBR 24.1%; mPFS 4.4 months mOS 7.3 months, PR 9 patients	The safety profile was found to be tolerable with dose reductions, and the absence of IHC analysis was the major shortcoming in the study design
Tusamitamab ravtansine	–	NCT02187848	I/II	92	Non-squamous NSCLC having moderate to high CEACAM5 expression	Heavily pretreated NSCLC	PR was observed in nearly half of the treated patients	The response observed was durable and long-term benefit was seen even after 1 year of treatment
Trastuzumab Emtansin	–	NCT02675829	II	18	NSCLC-AC with HER-2 mutations	Phase-IV or recurrent NSCLC previously treated with two lines of systemic therapies	mPFS 5 months; PR rate 44%	This trial provided proof-of-concept for targeting HER-2 mutation through ADCs in NSCLC patients
	–	–	II	22	HER2 exon 20 insertion mutations	Stage III/IV or post-operative recurrence of NSCLC-AC	DCR 52.4%; ORR 38.1%; mPFS 2.8 months; mOS 8.1 months; DOR 3.5 months	Safety profile was found to be safe and tolerable with limited occurrences of grade > 3 toxicities
	–	NCT02289833	II	49	HER-2 overexpression	Pretreated advanced NSCLC	ORR 20%; CBR 30%	Patients were divided into IHC2 + and IHC3 + cohorts, but no significant difference was found in the efficacy
Trastuzumab deruxtecan	–	NCT03505710	II	91	HER2 mutations	Refractory NSCLC	ORR 55%; mDoR 9.3 months; mOS 17.8 months; mPFS 8.2 months	Efficacy was observed even in patients with IHC grade 0
	DESTINY-Lung02 trial	NCT04644237	II	151	HER2 mutations	Pretreated NSCLC	ORR of 53.8% and 42.9% in the 5.4 mg/kg and 6.4 mg/kg cohort, respectively	A higher occurrence of AEs was observed in the 6.4 mg/kg cohort
A166	–	NCT03602079	I	35	HER2 positive or HER2 amplified tumors	Patients with solid tumors who had relapsed on previous lines of therapies	DCR 59%; SD 33%; PR 26%	Response to treatment was observed in doses between 3.6 mg/kg and 4.8 mg/kg
Patritumab deruxtecan	–	NCT03260491	I	57	EGFR mutations	Advanced/metastatic NSCLC previously treated with TKIs	ORR 39%; mPFS 8.2 months	This trial led to the approval of this ADC as a breakthrough therapy in patients who have relapsed following TKI therapy

*EGFR* epidermal growth factor receptor, *NSCLC* non-small cell lung cancer, *c-MET* c-Mesenchymal-epithelial transition factor-1, *ORR* overall response rate, *TKIs* tyrosine kinase inhibitors, *NSCLC-AC* NSCLC-adenocarcinoma, *NSCLC-SCC* NSCLC-squamous cell carcinoma, *mOS* median overall survival, *mPFS* median progression-free survival, *FRA* folate receptor α, *CR* complete response, *PR* partial response, *SD* stable disease, *DCR* disease control rate, *RP2D* recommended phase-II dose, *CBR* clinical benefit rate, *ICIs* immune checkpoint inhibitors, *IHC* immunohistochemistry, *CEACAM-5* carcinoembryogenic antigen-related cell adhesion molecule-5, *AEs* adverse events, *HER-2* human epidermal growth factor-2, *DOR* duration of response

*REGN5093-M114* is another ADC efficient for targeting the acquired MET-amplification driving the NSCLC tumor progression [29]. A phase I/II, open-label study (NCT04982224) is currently under investigation for evaluating the role of REGN5093-M114 in advanced NSCLC patients showcasing MET-overexpression [29]. Positive outcomes from this study can embark upon a detailed investigation of this molecule in MET-overexpressing NSCLC patients.

*MYTX-011* is one additional ADC working by diminishing MET amplification contributing to carcinogenesis. A phase 1 study (NCT05652868) is currently under investigation to assess the safety, tolerability, and preliminary effectiveness of MYTX-011 in patients with advanced, recurrent, or metastatic NSCLC [30]. Currently, ongoing clinical trials of various ADCs for the use in NSCLC are presented in Table 3.

**Table 3:** Currently ongoing trials evaluating ADCs in NSCLC

ADC name	NCT no	Phase	Patient characteristics	Intervention	Primary outcome	Secondary outcome
Trastuzumab Deruxtecan [DS-8201]	NCT04042701	1	Locally advanced/metastatic breast cancer or NSCLC with HER2-expression or mutation	Trastuzumab deruxtecan + pembrolizumab	DLT and ORR	PK parameters, DoR, DCR, PFS, TTR, OS
	NCT04644237	2	HER2 mutated NSCLC, which reoccurred or progressed during/after at least One regimen of prior anticancer therapy that contained a platinum-based chemotherapy drug	Trastuzumab deruxtecan alone	ORR	DoR, PFS, OS, DCR, PK parameters
	NCT05246514	2	HER2 mutant metastatic NSCLC, which progressed during/ after atleast one-line of treatment	Trastuzumab deruxtecan alone	ORR	DoR, PFS, OS, DCR, immunogenicity, frequency of AEs
	NCT05048797	3	Unresectable, locally advanced, or metastatic NSCLC harboring HER2 exon 19 or 20 mutations	Trastuzumab deruxtecan vs cisplatin, carboplatin, pemetrexed, pembrolizumab	PFS	ORR, DoR, OS, immunogenicity, PK parameters
	NCT04686305	1	Advanced or metastatic HER2 + NSCLC	Trastuzumab deruxtecan + durvalumab + cisplatin/carboplatin/pemetrexed	Frequency of AEs, SAEs	ORR, DCR, OS, PFS, PK parameters
MRG002	NCT05141786	2	HER2-mutated unresectable/metastatic NSCLC, which failed at least one prior line of a stand of care therapy	MRG002 alone	ORR	AEs, OS, PFS, DoR, PK parameters
Pertuzumab Zuvotolimod [SBT6050]	NCT04460456	1	Advanced solid tumors expressing HER2 [includes NSCLC]	SBT6050 alone and SBT6050 + pembrolizumab /cemiplimab	ORR, DoR, AEs	PK parameters, PFS, DCR, DoR
GQ1001	NCT04450732	1	HER2-positive advanced solid tumors [includes NSCLC]	GQ1001 alone	DLT, MTD	AEs, PK parameters, DoR, DCR, immunogenicity
Patritumab Deruxtecan [U3-1402]	NCT04619004	2	Metastatic or locally advanced NSCLC with an activating EGFR mutation, which progressed during/after at least 1 EGFR TKI and 1 platinum-based chemotherapy-containing regimen	Patritumab deruxtecan alone	ORR	DoR, PFS, DCR, TTR, OS, incidence of TEAE, SAEs
	NCT05338970	3	Metastatic or locally advanced EGFR mutated NSCLC, Which failed EGFR TKI therapy	Patritumab deruxtecan vs platinum-based chemotherapy	PFS	OS, PFS, ORR, DoR, DCR, TTR, CBR
	NCT04676477	1	Locally advanced or metastatic EGFR-mutated NSCLC	Patritumab deruxtecan + osimertinib	DLT, TEAE, SAEs	ORR, DCR, DoR, TTR, PFS, PK parameters
Telisotuzumab Vedotin [Teliso-V]	NCT05513703	2	Previously untreated MET amplified locally advanced/metastatic NSCLC	Telisotuzumab vedotin alone	ORR	DoR, DCR, PFS, OS
	NCT04928846	3	c-Met overexpressing NSCLC and have received no more than 1 line of prior systemic cytotoxic chemotherapy	Telisotuzumab vedotin vs docetaxel	PFS, OS	ORR, DoR, PFS
	NCT03539536	2	Previously treated c-Met + NSCLC and received no more than 2 lines of prior systemic therapy	Telisotuzumab vedotin alone	ORR, number of participants with AEs	DoR, DCR, PFS, OS
REGN5093-M114	NCT04982224	1 and 2	MET-overexpressing NSCLC	REGN5093-M114 alone	DLT, SAEs, TEAE, ORR	DoR, DCR, PFS, OS, immunogenicity, TTR
MYTX-011	NCT05652868	1	NSCLC with cMET overexpression /MET amplification/exon 14 skipping mutations with a current unmet medical need	MYTX-011 alone	DLT, number of participants with tumor response	PK parameters, DoR, DCR, TTR, PFS, OS
M1231	NCT04695847	1	Locally advanced or metastatic solid tumor, Which expressed or likely to expressed EGFR and MUC1, intolerant or refractory to standard therapy [includes NSCLC]	M1231 alone	OR, DoR, TEAE	PFS, DoR, PK parameters, OS
AZD9592	NCT05647122	1	Advanced solid tumor with EGFR mutation [includes NSCLC]	AZD9592 alone and in combination with osimertinib	ORR, DLT, SAEs	ORR, DCR, DoR, PFS, OS, PK parameters, immunogenicity
MRG003	NCT04838548	2	EGFR-positive advanced NSCLC	MRG003 alone	ORR	PFS, TTR, DCR, OS, DoR, AEs
Datopotamab deruxtecan [DS-1062]	NCT04526691	1	Previously treated or treatment-naive patients with advanced/metastatic NSCLC without actionable genomic alterations	Datopotamab deruxtecan + pembrolizumab with or	Number of participants with DLT, TEAE	OS, PFS, DoR, ORR, Anti-drug antibodies, PK parameters
	NCT05555732	3	Advanced or metastatic PD-L1 TPS < 50% non-squamous NSCLC with no prior therapy and without actionable genomic alterations	Datopotamab deruxtecan + pembrolizumab with or without platinum chemotherapy	PFS, OS	ORR, PFS, DoR, TTR, DCR, number of participants with TEAEs
	NCT05215340	3	Untreated advanced or metastatic high PD-L1 (TPS ≥ 50%) NSCLC without actionable genomic alterations	Datopotamab deruxtecan + pembrolizumab vs pembrolizumab	PFS, OS	ORR, PFS, TTR, DCR, number of participants with TEAE
	NCT04612751	1	Advanced or metastatic NSCLC without actionable genomic alterations	Datopotamab deruxtecan + immunotherapy With or without 4 cycles of carboplatin	Number of participants with DLT and TEAE	PFS, OS, DoR, TTR, DCR, PK parameters
	NCT04484142	2	Advanced or metastatic NSCLC with actionable genomic alterations, which progressed during/after targeted therapy and platinum-based chemotherapy	Datopotamab deruxtecan alone	ORR	DoR, PFS, OS, PK parameters
	NCT04656652	3	Previously treated advanced or metastatic NSCLC with or without Actionable genomic alterations	Datopotamab deruxtecan vs Docetaxel	PFS, OS	DoR, TTR, DCR, ORR, PK parameters
	NCT03401385	1	Trop-2 expressing advanced solid tumor [includes NSCLC]	Datopotamab deruxtecan alone	Number of participants with DLT, AEs	PK parameters
	NCT04940325	2	Metastatic, unresectable NSCLC, Which progressed on 1, but not more than 3 previous standard therapies	Datopotamab deruxtecan alone	ORR	DoR, PFS, incidence of AEs, CBR
	NCT05460273	1 and 2	Adult Chinese participants with advanced or metastatic solid tumors [includes NSCLC]	Datopotamab deruxtecan alone	ORR	PFS, OS, PK parameters, DoR, DCR, immunogenicity
Sacituzumab Govitecan [IMMU-132]	NCT05089734	3	Advanced or metastatic NSCLC, which progressed during/after platinum-based chemotherapy and anti-PD-1/PD-L1 immunotherapy	Sacituzumab govitecan vs docetaxel	OS	PFS, ORR, DCR, DoR
	NCT05186974	2	Advanced or metastatic NSCLC without actionable genomic alterations, with no prior systemic treatment for metastatic NSCLC	Sacituzumab govitecan + pembrolizumab or sacituzumab govitecan pembrolizumab + a platinum agent	ORR, percentage of participants experiencing DLT	OS, PFS, DoR, DCR, percentage participant experiencing TEAE
	NCT05609968	3	Metastatic NSCLC with PD-L1 TPS ≥ 50%	Sacituzumab govitecan vs pembrolizumab	PFS, OS	OR, DoR, number of participants experiencing AEs
	NCT03964727	2	Metastatic solid tumors [includes NSCLC]	Sacituzumab govitecan alone	ORR	ORR, PFS, DoR, CBR, OS
SKB264	NCT05351788	2	Advanced or metastatic NSCLC	SKB264 + KL-A167 with or without chemotherapy	ORR, incidence, and severity of AEs	PFS, DoR, DCR, PK parameters
	NCT04152499	1 and 2	Locally advanced unresectable/metastatic solid tumors, refractory to available standard therapies [includes NSCLC]	SKB264 alone	MTD, RDE, ORR	DLT, ORR, PFS, DoR, OS
Tusamitamab ravtansine [SAR408701]	NCT04524689	2	CEACAM5 positive advanced/metastatic non-squamous NSCLC	Tusamitamab ravtansine + Pembrolizumab and Tusamitamab ravtansine + Pembrolizumab + platinum-based chemotherapy and with or without pemetrexed	Drug-related DLT	ORR, number of participants with TEAE, SAEs
	NCT05245071	2	Non-squamous NSCLC with negative or moderate CEACAM5 expression	Tusamitamab ravtansine alone	ORR	PFS, DoR, DCR, Incidence of participants with TEAE,
	NCT04154956	3	Previously treated CEACAM5 positive metastatic NSCLC	Tusamitamab ravtansine alone	PFS, OS	ORR, DoR, HRQOL, number of participants with TRAEs and SAEs
Tisotumab vedotin [TF-011-MMAE]	NCT03485209	2	Locally advanced or metastatic solid tumors, which progressed during or after their most recent systemic therapy [includes NSCLC]	Tisotumab vedotin alone and in combination pembrolizumab with carboplatin or cisplatin	ORR	DCR, DoR, TTR, PFS, OS, AEs

*NSCLC* non-small cell lung cancer, *HER-2* human epidermal growth factor-2, *DLT* dose-limiting toxicity, *ORR* overall response rate, *PK* pharmacokinetics, *DoR* duration of response, *DCR* disease control rate, *PFS* progression-free survival, *TTR* time in therapeutic range, *OS* overall survival, *AEs* adverse events, *EGFR* epidermal growth factor receptor, *TKIs* tyrosine kinase inhibitors, *MTD* maximum tolerated dose, *TEAEs* treatment-emergent adverse events, *SAEs* serious adverse events, *PD-L1* programmed cell death ligand, *ADC* antibody–drug conjugates, *CBR* clinical benefit rate, *TRAEs* treatment-related adverse events, *HRQOL* health-related quality of life, *CEACAM-5* carcinoembryogenic antigen-related cell adhesion molecule-5, *RDE* recommended dose for expansion, *TPS* tumor proportion score, *c-MET* c-mesenchymal-epithelial transition factor-1

#### TROP-2

Human trophoblast cell-surface antigen 2 (TROP-2) is a promising molecular biomarker owing to its high expression in numerous epithelial carcinomas including NSCLC [31]. Encoded by the tumor-associated calcium signal transductor (Tacstd2) gene, TROP-2 is a transmembrane glycoprotein and an intracellular calcium signal transducer with downstream signaling supporting cell survival, promoting proliferation and migration along with invasion in tumor cells [32]. Overexpression of TROP-2 is more commonly observed in NSCLC-SCC than in lung adenocarcinoma which is independent of sex, histologic grade, and pathologic stage [33]. The presence of this overexpression is associated with poor prognosis and lung cancer-specific mortality. Formerly mentioned substantiations have been derived from a study investigating TROP2 as a potential prognostic marker and therapeutic target. The in-vitro study included human bronchial epithelial cells and NSCLC cell lines. The NSCLC cell lines were cultured in RPMI 1640 (Gibco, Invitrogen Life Technologies, Carlsbad, CA, USA). The tissue samples and adjacent normal tissue samples were obtained post-retrieval of informed consent from 20 patients. These samples were further snap-frozen in liquid nitrogen followed by immunohistochemical staining using the standard streptavidin-biotin-peroxidase complex method. The construction of TROP-2 silenced and overexpressing cell lines was practiced after which the real-time quantitative reverse transcription PCR, western blot, wound healing assay, cell proliferation assay, transwell migration assay, and flow cytometry assay were performed. The results reported TROP-2 overexpression which correlated significantly with clinical stage, differentiation, lymph node metastasis, and distant metastasis [34]. The study affirmed TROP-2 as an oncogene in lung adenocarcinoma.

At present, there are three ADC molecules (Datopotamab deruxtecan, Sacituzumab govitecan, and SKB264) under investigation, which can identify and target TROP-2.

*Datopotamab deruxtecan*, otherwise known as Dato-DxD and DS-1062a, was evaluated in the TROPION-PanTumor01 (NCT03401385) trial. The results of the dose escalation/expansion phase in patients with advanced NSCLC exhibited a disease control rate (DCR) of 87%, ORR of 21%, and median PFS of 8.2 months along with a manageable safety profile. However, interstitial lung disease (ILD) was observed in 8 patients. The 6 mg/kg dose of Dato-DXd as monotherapy is currently under phase-III trial [35].

TROPION-Lung02 (NCT04526691) trial is currently evaluating the combination of Dato-DXd and Pembrolizumab with or without chemotherapy in treatment naïve or previously treated subjects with metastatic/advanced NSCLC [36]. Evaluating this combination in the treatment of naïve patients is crucial and can significantly improve the treatment strategies for patients with advanced NSCLC in the future.

Another phase-III trial called TROPION-Lung08 (NCT05555732) is assessing the superiority of Dato-DXd combined with Pembrolizumab compared to Pembrolizumab alone in treatment naïve patients with metastatic/advanced NSCLC without actionable genomic alterations. Nearly 740 participants will be enrolled whose tumor expresses high PD-L1 expression (> 50%). The primary endpoints of the study are PFS and OS [37].

The majority of discussed trials above focus on the treatment of naïve patients with advanced NSCLC. Affirmative outcomes from these studies have the potential to alter the treatment landscape of patients suffering from advanced NSCLC, by incorporating the ADCs and immunotherapeutic agents in the earlier lines of therapy where the odds of achieving improved prognostic outcomes are significantly higher.

*Sacituzumab govitecan-hziy* (SG) has proven its efficacy and superiority over other therapeutic modalities in various cancers including metastatic triple-negative breast cancer (mTNBC). Following the encouraging data from numerous clinical studies, SG received accelerated approval from the FDA for treatment in mTNBC. At present, several studies are evaluating the safety and efficacy of the same in patients with NSCLC. The IMMU-132-01 trial assessed the safety and antitumor efficacy of SG in patients with advanced epithelial cancers which included the NSCLC cohort. The study enrolled patients with metastatic NSCLC who were heavily pre-treated with other therapeutic modalities including ICIs. The patients were treated with 8 mg/kg or 10 mg/kg dose of SG on days 1st and 8th of a 21-day cycle. The median response duration from the treatment was 6 months along with an ORR of 16.7% and a clinical benefit rate (CBR) of 24.1%. Partial response (PR) was observed in 9 patients and complete response (CR) was not observed at all. Additionally, median PFS and median OS were 4.4 months and 7.3 months, respectively. The most commonly observed adverse events (AEs) were neutropenia and diarrhea, which were generally tolerable with dose reductions. The patients who progress after two or more lines of therapy in the metastatic setting generally face dismal prognostic outcomes and have very few treatment options apart from palliative care. The encouraging results from this trial hint toward the addition of SG in the treatment regimen for the heavily pre-treated patients with advanced disease. One significant limitation of this trial was the absence of immunohistochemistry (IHC) analysis to assess the TROP-2 positivity [38]. Assessing the impact of TROP-2 expression on the antitumor activity of SG can aid in selecting the patient population that can benefit the most from the treatment. Thus, future clinical studies evaluating SG should aim to focus on correlating the TROP-2 expression with the antitumor responses of the drug.

#### CEACAM-5

Carcinoembryogenic antigen-related cell adhesion molecule-5 (CEACAM-5) belongs to the CEACAM group with highly glycosylated proteins possessing a typical N-terminal variable Ig domain and is located on the cell surface [39]. CEACAM-5 is found to have an inhibitory action on p38 activity which regulates multiple complex biological processes such as cell proliferation, cell differentiation, death, migration, and invasion [40]. CEACAM-5 has been noted as a tumor biomarker and recurrence detector in cancer patients, especially those suffering from colorectal carcinoma [41]. Pre-clinical studies had been designed to unveil CEACAM-5 utility in NSCLC. The *in vitro* and *in vivo* study performed focusing on the formerly mentioned aim investigated the role of CEACAM-5 in NSCLC progression [42]. Tumor samples, post-isolation were fixed with paraformaldehyde, embedded in paraffin, and cut into 5 µm sections. The following procedures included deparaffinization, rehydration, and incubation for 10 min. Blocking of samples was undertaken using 1% normal goat serum which later was followed by treatment with CEACAM-5 antibody. After immunohistochemistry, several other tests such as real-time quantitative reverse transcription PCR, immunoblotting, cell viability and colony formation assay, wound healing assay, and animal experiments on nude mice were also performed. The study presented findings in the form of an over-expression of CEACAM-5 in human NSCLC tissues and cells. The expression was also found to be correlated with the clinicopathological status of NSCLC patients. The expression of CEACAM-5 and its role in driving the NSCLC oncogenesis was also verified and the *in vitro* and *in vivo* study results demonstrated the involvement of the p38-Smad2/3 signaling pathway [39]. Hence, this biomarker was deduced to be a potential therapeutic target for the treatment of NSCLC.

The first-in-human study (NCT02187848) of a novel ADC *Tusamitamab ravtansine* (SAR408701) demonstrated a favorable safety profile along with ORR of 22.7% in patients with heavily pretreated solid tumors with more than 50% tumor cells expressing CEACAM5 [43]. Building on this premise, this ADC was further evaluated in 92 patients with non-squamous NSCLC having moderate to high CEACAM5 expression. The results demonstrated PR in nearly half of the treated patients even after more than 1 year of treatment [44]. These observations hint toward a durable response and long-term benefit even in previously relapsed patients, which is highly uncommon in patients with advanced malignancies.

#### EGFR

EGFR, is a tyrosine kinase receptor belonging to the ERBB family [45]. When an extracellular ligand binds to EGFR, it triggers the receptor to undergo homo- or heterodimerization, leading to phosphorylation of tyrosine kinase sites within the cytoplasm. This, in turn, initiates various intracellular pathways, including the PI3K/AKT/mTOR and RAS/RAF/MAPK which control cell proliferation and metastasis and prevent apoptosis [46]. The majority of the EGFR mutations in NSCLC have been identified in exons 18–21 of the tyrosine kinase domain of the receptor, which encode for a portion of the EGFR kinase domain. EGFR 19 deletion or an EGFR L858R point mutation is frequently observed in NSCLC cases [47]. The less prevalent EGFR mutations include G718X, S768I and L861Q. A special type of EGFR mutation—in-frame insertion in exon 20 (T790M)—occurs in 3–7% of NSCLC cases. EGFR mutations are commonly found in females, never smokers or are light smokers, those of Asiatic origin, and those with NSCLC-AC (10–35%) that have advanced to a locally advanced or metastatic stage (Stage III–IV) [48]. The analysis of EGFR mutations is primarily performed using direct sequencing of DNA and real-time polymerase chain reaction (RT-PCR)-based assays. Both methods demonstrate high performance in detecting these mutations in tissues that have been fixed in formalin and embedded in paraffin. Another method for detecting EGFR mutations is fragment length analysis, which is capable of identifying insertions or deletions but cannot detect point mutations in the EGFR gene [49]. The standard approach for treating advanced or metastatic disease in patients with EGFR-mutant tumors is the use of first- or second-generation EGFR TKIs as initial therapy. The first-generation TKIs, such as gefitinib and erlotinib, are reversible inhibitors that demonstrate strong inhibitory activity against both the wild-type EGFR and sensitive mutant EGFR. In contrast, the second-generation TKIs, including afatinib and dacomitinib, are irreversible inhibitors. Studies have shown that these TKIs lead to a significantly higher ORR and longer PFS compared to first-generation TKIs. However, there is no significant change in OS with the use of second-generation TKIs [50]. The use of TKIs is limited due to dose-limiting toxicity and the development of resistance in patients [51]. In the treatment of NSCLC patients who are T790M-positive and resistant to earlier-generation TKIs, the third-generation EGFR-TKI osimertinib has become the standard approach [52]. Osimertinib is associated with relatively milder gastrointestinal and skin toxicity compared to previous TKIs [53]. EGFR-mutant tumors, on the other hand, do not respond well to checkpoint inhibitors targeting PD-1 or PD-L1. Therefore, the use of immunotherapy alone is not recommended [54]. Due to the toxicity and development of resistance associated with conventional therapies, the use of ADCs is gaining interest.

*MRG003* is currently being investigated for the treatment of EGFR-positive advanced NSCLC. A phase II study is underway to evaluate the effectiveness of MRG003 in patients with EGFR-positive advanced NSCLC. This study aims to gather valuable information about the efficacy, safety, and overall therapeutic potential of MRG003 in treating advanced NSCLC patients with EGFR-positive tumors [55].

#### HER-2

The human epidermal growth factor-2 (HER-2) gene, also known as ErbB2, is a proto-oncogene belonging to the ERBB receptor tyrosine kinase family. HER2 alterations can occur in three forms: gene amplification, overexpression, and point mutations [56]. These mutations are more commonly observed in lung adenocarcinomas, particularly in females, Asians, and individuals who are either never smokers or light smokers [57]. The highest prevalence of these mutations is observed in stage III-IV NSCLC patients. Exon 20-point mutations, namely L755S and G776C, are the most frequently observed HER2 mutations, while other less common mutations include G660D, R678Q, E693K, and Q709L. HER2 mutations can be detected through RT-PCR or gene sequencing methods like NGS. HER2 amplification is identified using FISH analysis, enzyme-linked immunosorbent assay (ELISA), or NGS, while HER2 overexpression is detected through IHC [58]. Treatment options for HER2-mutant tumors include HER2-TKIs, anti-HER2 antibodies, and ADCs. HER2-TKIs are associated with safety concerns and tolerability issues [56]. ADCs are gaining attention due to their limited toxicity and high tumor selectivity [59].

*Trastuzumab Emtansin (T-DM1) *is an ADC approved by the FDA for patients with HER-2-positive breast cancer. However, the data for the same in the NSCLC have not been conclusive yet.

A phase-II basket trial (NCT02675829) enrolled 18 patients with adenocarcinoma subtype whose tumors expressed HER2 mutations. These subjects were treated with 3.6 mg/kg T-DM1 every 3 weeks. The results showcased a median PFS of 5 months and a partial response rate of 44%. Commonly observed toxicities were elevation in hepatic transaminase enzymes, thrombocytopenia, and infusion-related reactions. After nearly a decade of unfavorable results from studies targeting HER2 in lung cancer, these results offered several essential insights and embarked upon further trials of ADCs targeting HER2 in lung cancers [60].

A phase II clinical trial (NCT02289833) by Peters et al. evaluated T-DM1 in patients with pretreated advanced NSCLC showcasing HER-2 overexpression. The 49 enrolled patients in this study were divided into two cohorts based on IHC2+ and IHC3+ expression. The results demonstrated favorable clinical outcomes in the IHC3+ cohort compared to the IHC2+ cohort. In the IHC3+ cohort, reported ORR and CBR were 20% and 30%, respectively. However, there was no significant difference between the mPFS and mOS observed in both cohorts, which indicates that IHC grade is not a definitive predictor of efficacy.

Following encouraging outcomes from these trials, T-DM1 was given a category 2A recommendation from the National Comprehensive Cancer Network (NCCN) for the treatment of patients with advanced NSCLC harboring HER2 mutations [61].

*Trastuzumab deruxtecan* (T-DXd) is a topoisomerase-I inhibitor that is a widely utilized HER2 targeting agent in various malignancies as it is approved by the FDA for use in metastatic or advanced HER2-positive breast cancer and in advanced HER2-positive gastric cancer which is trastuzumab refractory.

A phase-II clinical trial (NCT03505710) by Li et al. evaluated the clinical outcomes of T-DXd in 91 patients with refractory HER2 mutant NSCLC. The results revealed an ORR of 55%, mDoR of 9.3 months, mOS of 17.8 months, and mPFS of 8.2 months. The safety analysis showed the occurrence of grade > 3 AEs in 46% of patients and the occurrence of ILD in 26% of patients. Moreover, ILD resulted in mortality in 2 patients. An intriguing observation from this study was that the efficacy of T-DM1 was observed in even those patients who had HER2 mutation but the IHC grade was 0 (lack of detection of HER2 expression). These observations might be the result of activating HER2 mutations leading to the enhancement of the intracellular uptake of ADC-HER2 receptor complex and receptor internalization [62]. However, further studies are mandatory to procure insights regarding the HER2 expression and clinical efficacy of ADCs.

The DESTINY-Lung02 trial (NCT04644237) compared the safety and efficacy profile of 5.4 mg/kg and 6.4 mg/kg of T-DXd in patients with pretreated NSCLC with HER2 mutation. The interim analysis of the data was presented at the 2022 European Society of Medical Oncology (ESMO) meetings. The results demonstrated ORR of 53.8% and 42.9% in the 5.4 mg/kg and 6.4 mg/kg cohort, respectively. Analysis of the safety data showed that the treatment-emergent adverse events (TEAEs) were higher in the 6.4 mg/kg cohort, with 14% of the patients in this cohort developing ILD. Thus, these results exhibited encouraging clinical activity of T-DXd in patients with NSCLC alongside a promising safety profile in the 5.4 mg/kg cohort. The encouraging results from this study led to the FDA approval of T-DXd at 5.4 mg/kg dose in patients with pretreated HER2-mutant NSCLC [63].

As reported from the previously discussed studies, the occurrence of ILD remains a huge challenge while treating patients with T-DXd. Thus, it is crucial to identify the potential risk factors for developing the ILD as it has proven to be fatal in many patients. Additionally, it was reported that the incidence of ILD was higher in patients with lung cancer when compared to breast cancer or gastric cancer cases. This can be attributed to the preexisting damage to the lung tissue owing to smoking, radiation, or surgery leading to compromised pulmonary function in patients with lung cancer. Hence, identifying the mechanism and carrying out routine surveillance of these patients can aid in reducing the mortality caused by ILD [59].

*A166* is an ADC that was evaluated in a phase I study (NCT03602079), as monotherapy in patients with solid tumors who had relapsed on previous lines of therapies. The results showed promising clinical efficacy with a manageable safety profile. The doses between 3.6 mg/kg and 4.8 mg/kg demonstrated a response to treatment, with DCR, SD, and PR observed in 59%, 33%, and 26% of patients, respectively [64].

#### HER-3

HER3, also known as ErbB3, is a member of the EGFR receptor family and is located on chromosome 12q13. Upon binding with ligands neuregulin 1 or 2, HER3 can undergo homodimerization or heterodimerization with HER2, leading to the activation of downstream signaling pathways that promote cell survival and proliferation [65]. Overexpression of HER3 in patients with NSCLC has been associated with increased metastasis, reduced survival, and resistance to anti-EGFR/HER2 therapies. Therefore, HER3 has emerged as a potential therapeutic target in cases with EGFR and HER2 mutations [66]. HER3 overexpression is commonly observed in patients with locally advanced or metastatic adenocarcinoma, often accompanied by overexpression of EGFR and/or HER2. Detection of HER3 overexpression can be achieved through NGS, IHC, PCR, or FISH analysis [67]. Currently, there are no approved medications specifically targeting HER3 mutant NSCLC. However, various mono- and bispecific monoclonal antibodies, ADCs, and small-molecule inhibitors that target HER3 are being investigated as potential treatment options.

*Patritumab deruxtecan* (HER3-DXd/U3-1402) is an ADC currently undergoing phase I clinical trials. In a phase I trial (NCT03260491) involving 57 subjects with advanced/metastatic NSCLC with EGFR mutation who had received TKIs, HER3-DXd was administered at a dose of 5.6 mg/kg every 3 weeks. The results showed an ORR of 39% mPFS of 8.2 months. Hematological toxicities were the most commonly encountered grade 3 treatment-related adverse events. Currently, therapeutic options are limited for patients with EGFR mutations who have relapsed on TKI therapy. The promising results from this study suggest that HER3-DXd could be a potential therapeutic option for TKI-resistant cases. Based on these findings, the FDA granted BTD to HER3-DXd for NSCLC patients with locally advanced/metastatic disease and EGFR mutation with disease progression following 3rd generation TKI therapy [68].

The dose expansion phase of the same trial enrolled NSCLC patients without EGFR mutations who had received prior treatment with platinum-based chemotherapy alone or in combination with immunotherapy. Out of the 47 enrolled patients, 17 had KRAS, ALK, NRAS, and ROS genomic alterations. The trial results demonstrated a DOR of 5.7 months, mPFS of 5.4 months, and ORR of 28%. Confirmed responses were observed in 23% of patients without any genomic alterations and in 35% of patients with actionable genomic mutations [69]. This trial highlights the potential of ADCs in treating patients both with and without actionable genomic alterations. These findings have the potential to reshape the future of cancer treatment paradigms.

## Challenges associated with ADC monitoring and administration

Implementation of ADCs in the therapeutic regimen is contracted with several challenges that limit their prescription and accompany decision-making-related confusion to the prescribing clinicians [70]. Ensuring the stability of drug payload while the molecule is circulating in the bloodstream is very critical. Release of this payload only after it reaches the target checkpoint is necessary to surpass off-target toxicities and inadequate efficacy. Moreover, a thorough pharmacokinetic profile of ADCs must be derived in order to predict the degree of accumulation of ADCs in the tumor tissues sparing the healthy cancer-free cells [71, 72]. Another challenge is put forth considering the optimization of dosage regimen and schedule for ADCs. Individual patient variability, payload pharmacokinetics, tumor types, and staging-related factors may need additional consideration while employing ADCs in therapy [73, 74]. Additionally, the mAbs assembled to form the ADCs can hold immunogenic potential which further triggers the development of antibodies against the ADC. This immune response can decrease the effectiveness and affect the safety aspects of ADCs introducing challenges such as allergic reactions or suboptimal responses [75].

Taking into consideration the imaging and diagnostics aspects, obstacles are faced in marking the course of ADC reaching the tumor site. Real-time imaging may assist in determining whether the intended tumor site is targeted by ADCs or not. Moreover, techniques for quantification of ADC concentrations and drug-antibody ratio need to be developed to ensure utmost precision and reliability [71]. Despite numerous advances, ADC implementation-related decisions are reconsidered due to the costs of these molecules. The development and production of ADCs can be expensive leading to higher treatment costs which limit their applicability. As a consequence of this reason, ADCs are under-utilized in healthcare systems with limited resources [76]. Addressing these concerns requires the involvement of multiple disciplines and the expertise of researchers, regulatory agencies, clinicians, and pharmacists. Persistent investigational campaigns exploring the analytical techniques and innovation in ADCs can help in improving the monitoring and patient outcomes.

### Nano-technological interventions and advanced drug delivery platforms

Nanomedicine is a formulation-based approach to drug delivery that involves the encapsulation of cargo drugs in nanoparticles [77]. These nanoparticles can be made from organic substances, such as liposomes, dendrimers, and nanogels, or inorganic substances, such as gold nanoparticles, paramagnetic lanthanide ions, and quantum dots [78]. Antibodies are conjugated to nanoparticle-encapsulated drugs to form antibody-conjugated nanoparticles (ACNPs), This combines the benefits of nanoparticles with monoclonal antibodies to give large drug loads at the tumor location while improving selectivity and delivery efficiency and minimalizing drug leakage [79]. ACNPs offer several advantages over ADCs, such as a higher drug-to-antibody ratio than ADCs, which ensures the internalization of a higher concentration of the drug. It can also be designed to release the drug in a controlled manner, which can reduce toxicity and improve efficacy [80]. Additionally, it can be loaded with a wider range of chemotherapeutic drugs than ADCs. Finally, ACNPs have a large number of antibodies on their surface, which can produce a multivalency effect that induces hyperclusterization and increases avidity[(80]. Several studies have investigated the use of ACNPs for lung cancer, including a study of cetuximab and doxorubicin-loaded dextran-coated Fe3O4 magnetic nanoparticles found that the nanoparticles were able to target and suppress the proliferation of NSCLC cell line A549 more effectively than nanoparticle-encapsulated doxorubicin alone [81]. A study of a nanoparticle co-delivering PLK1 inhibitor (volasertib) and PD-L1 antibody found that the nanoparticles were able to reduce the effective dosages of volasertib and PD-L1 antibody by 5-fold in a metastatic lung tumor model, thus overcoming the issue of dose-related toxicity [82]. A study of P-glycoprotein antibody-conjugated paclitaxel PEG-coated immunoliposomes (Pab-PTX-L) found that the nanoparticles were more effective at killing paclitaxel-resistant lung cancer A549/T cells than paclitaxel alone [83]. Despite their many benefits, the use of ACNPs for cancer therapy is limited by several challenges, including the design and production of targeted nanoparticles with favorable biocompatibility, pharmacokinetics, in vivo targeting efficacy, and cost-effectiveness; the lack of phase 3 clinical data; and the limited understanding of ACNP delivery and interactions in the human body.

### Future perspectives

The therapeutic profile of ADCs has shown noteworthy efficiency in NSCLC clinical trials, still, more space is left to be explored for further improvements. To achieve new breakthroughs, upgrades and refinement in the structural backbone of ADC may optimize the antitumor activity and minimize toxicity.

Delivery of potent ADC response requires accurate target binding with adequate internalization and appropriate trafficking for payload uncoupling and retention. All these individual processes may also transform into causes of ADC resistance. The proteomic investigation addresses payload retention as one of the critical steps in reinforcing ADC potential owing to the identification of upregulation of drug efflux pumps in T-DM1-resistant cell lines [84]. Moreover, the pre-clinical NSCLC studies report loss of SLC46A3 expression and increased expression of FOXO3α as responsible mechanisms for acquired resistance to DM1 and taxane-based ADC payloads respectively. It is unclear whether the resistance results from secondary mutations in the gene encoding target antigens and lysosome pathways or due to the shedding of target antigens from the cellular surface [85]. Additionally, the contribution of epigenetic mechanisms in the regulation of the expression of ADC targets is not explored extensively and demands intensive investigation.

Another limitation of ADC is related to their safety aspects as numerous clinical trials report unexpected dose-limiting toxicities (DLTs). Although ADCs are target-based therapy alternatives, it is possible for the payloads to detach and circulate in the bloodstream or leak out from the target cells [7]. To illustrate, the design of CX-2009 and XMT-1536 has been modified to overcome the narrow therapeutic index issues. Masking of the antigen-binding region of CX-2009 by a peptide is adopted and this is cleaved by proteases present in TME, which limits the on-target toxicity in healthy cells [86]. An instance observed related to this limitation is presented by Trastuzumab deruxtecan targeting HER2 tumor antigen. Incidences of ILD have been observed in patients receiving T-DXd which led to a search for potential risk factors contributing to its development [59].

ADC requires the expression of cell surface targets to exert their antitumor activity and the identification of these targets in a clinical setting has presented certain challenges. Tumors are small and heterogeneous specimens collected through fine needle aspiration biopsy or transbronchial biopsy. This specimen may not act as a reliable representative of the entire tumor which possibly leads to over- or underestimation of the presence of antigenic targets of ADC [74]. Additionally, it has become necessary to identify if higher target expression may facilitate better and more specific responses to ADCs in target-positive cancer settings. This need has raised the cause of clinical trials demonstrating ADC responses in tumors lacking surface antigen expression questioning specificity characteristic of an ADC. Another field of investigation for ADC is patient selection. For instance, the success of TROP2 ADCs confirmed that ADCs are not only functioning for oncogene-driven lung cancer and the antigen expression is not one of the key factors required to achieve optimal ADC responses. The antigen expression levels are continuously variable and this raises a dilemma whether pre-screening is required for patients before excluding them from the treatment regimen based on the presence of an antigen in particular amounts. Optimal inclusion parameters for enrolling patients in clinical studies evaluating ADCs are uncertain while some ongoing trials have opted for a pre-screening stage to recruit only those patients expressing specific ADC targets. Some clinical studies limit the inclusion criteria to tumor types positive for a specific level of high target expression.

Combination therapies have altered the basic ADC prescription as EGFR TKIs increase HER3 expression in NSCLC which can be easily detectable by the ADC. This instance provides a rationale for combining EGFR TKIs with HER3-targeted ADCs [87]. Another synergistic combination strategy is demonstrated by ADC combined with immunotherapy. This combined prescription promotes immunogenic cell death and ADCs can be intrinsically modified to enhance antibody-dependent cytotoxicity. Immunotherapy agents increase antitumor immunity induced by ADCs through acceleration of cell-mediated tumor recognition and immune effector function [88]. Alike EGFR TKIs, immunotherapy agents also increase the cell surface expression of target antigens on the tumor surface which allows for enhanced antibody-antigen coupling leading to improved antigen turnover and target degradation. Synergism was observed when ADCs were combined with ICIs or selective HER2 TKIs in preclinical studies [89]. This shifted the ADC monotherapy approach towards combination therapy giving futuristic prospects for ADC prescription. However, a major shortcoming was contracted for this combination strategy in the form of the occurrence of pneumonia when ADCs were given with ICIs. This raises a concern that this combination therapy may lead to pulmonary toxicity and hence, demands careful monitoring during administration.

Furthermore, multiple strategies are investigated to improve the current profile of ADC. One of the approaches involved the design of bispecific ADCs targeting two distinct tumor-associated antigens to enhance tumor-specificity and internalization. Biparatopic ADCs capable of targeting two different epitopes of the same target showed superior efficacy than single-target ADCS. To illustrate, HER2- targeted biparatopic ADC reported increased HER2 clustering and lysosomal degradation compared to trastuzumab and a more efficacious antitumor response than T-DM1 [90]. One of the approached introduced to combat resistance problems included dual payload ADCs which cleverly use two separate payloads in a controlled fashion to deliver a highly potent cytotoxic response. An anti-HER2 ADC built on this strategy consists of monomethyl auristatin E (MMAE) and monomethyl auristatin F (MMAF) and this ADC has resulted in promising efficacy in preclinical models [91]. Alternatives to cytotoxic agents incorporated as payloads are also under the development phase. Payloads consisting of immunotoxins and Bcl-2 inhibitors are not cytotoxic and function via induction of apoptosis. An example of one such ADC is ABBV-155 which targets the B7-H3 antigen and has clezutoclax payload that induces apoptosis by inhibiting Bcl-XL, rather than causing direct cytotoxicity [92]. Some other novel strategies include the conjugation of chemotherapy payloads directly to PD-1 antibodies, small molecule-based conjugates to improve drug delivery and potentiate blood–brain barrier (BBB) penetration, and aiming for extracellular matrix proteins to attack dense stroma that impedes drug delivery [93]. C’Dot Drug conjugates (CDCs) are assembled by ultra-small 6 nm diameter C’Dot silica nanoparticles which have the carrying capacity of 80 copies of payload. CDCs can carry up to 10 times more high-affinity targeting entities than ADCs, possess lower immunogenicity, and have less off-target interactions. These are extremely stable conjugates and hold high penetrability even across BBB. This concludes CDCs as a therapy reservoir for combating brain metastasis observed in advanced NSCLC settings [92].

## Conclusion

Following decades of extensive research and identification of various oncogenic drivers, NSCLC has become a prototype disease for precision oncology. The utilization of targeted immunotherapies was met with several challenges mainly the development of resistance and subsequently progression of disease. ADCs are innovative and novel therapeutic agents providing higher target specificity which reduces the off-target toxicities encountered with conventional chemotherapeutic agents. In patients suffering from advance stage NSCLC, where treatment options are limited, ADCs have shown promising clinical outcomes. ADCs namely telisotuzumab vedotin, trastuzumab deruxtecan, and patritumab deruxtecan have received FDA approvals in heavily pretreated patients with NSCLC; however, the role of ADCs in treatment naïve patients in early-stage settings are yet to be explored. A few clinical trials have demonstrated the potential of ADCs in targeting low-expressing tumors which widens the therapeutic horizon of these novel entities. Thus, designing clinical studies with a focus on correlating clinical efficacy with biomarker expression should be prioritized. Additionally, combining ADCs with immunotherapy and targeted therapy agents has shown encouraging results in several early-phase trials, which should be further explored to develop evidence-based recommendations in NSCLC patients. The utilization of ADCs is associated with several challenges such as the development of resistance as a result of target internalization, payload retention, and efflux mechanisms. Understanding and overcoming these hurdles can aid in the effective utilization of ADCs in routine clinical settings. Despite the challenges, the ADCs hold the potential to unveil a new era of precision oncology in NSCLC.

## Data Availability

Not applicable. Data sharing is not applicable to this article as no datasets were generated or analyzed during the current study.

## Abbreviations

NSCLC: Non-small cell lung cancer
ADCs: Antibody-drug conjugates
TKIs: Tyrosine kinase inhibitors
EGFR: Epidermal growth factor receptor
PD-1/PD-L1: Programmed cell death ligand-1
CTLA-4: Cytotoxic T-lymphocyte-associated protein 4
mAb: Monoclonal antibodies
FDA: Food and Drug Administration
HDF: Hepatocyte growth factor
NGS: Next-generation sequencing
FISH: Fluorescence *in-situ* hybridization
OS: Overall survival
PFS: Progression-free survival
TROP-2: Human trophoblast cell-surface antigen 2
ILD: Interstitial lung disease
